# The Symbiosis between *Lophelia pertusa* and *Eunice norvegica* Stimulates Coral Calcification and Worm Assimilation

**DOI:** 10.1371/journal.pone.0058660

**Published:** 2013-03-11

**Authors:** Christina E. Mueller, Tomas Lundälv, Jack J. Middelburg, Dick van Oevelen

**Affiliations:** 1 Department of Ecosystem Studies, Royal Netherlands Institute for Sea Research (NIOZ-Yerseke), Yerseke, The Netherlands; 2 Sven Lovén Centre for Marine Sciences, Tjärnö, University of Gothenburg, Strömstad, Sweden; 3 Department of Earth Sciences, Geochemistry, Utrecht University, Utrecht, The Netherlands; Bangor University, United Kingdom

## Abstract

We investigated the interactions between the cold-water coral *Lophelia pertusa* and its associated polychaete *Eunice norvegica* by quantifying carbon (C) and nitrogen (N) budgets of tissue assimilation, food partitioning, calcification and respiration using ^13^C and ^15^N enriched algae and zooplankton as food sources. During incubations both species were kept either together or in separate chambers to study the net outcome of their interaction on the above mentioned processes. The stable isotope approach also allowed us to follow metabolically derived tracer C further into the coral skeleton and therefore estimate the effect of the interaction on coral calcification. Results showed that food assimilation by the coral was not significantly elevated in presence of *E. norvegica* but food assimilation by the polychaete was up to 2 to 4 times higher in the presence of the coral. The corals kept assimilation constant by increasing the consumption of smaller algae particles less favored by the polychaete while the assimilation of *Artemia* was unaffected by the interaction. Total respiration of tracer C did not differ among incubations, although *E. norvegica* enhanced coral calcification up to 4 times. These results together with the reported high abundance of *E. norvegica* in cold-water coral reefs, indicate that the interactions between *L. pertusa* and *E. norvegica* can be of high importance for ecosystem functioning.

## Introduction

In the North East Atlantic, the scleractinian cold-water coral *Lophelia pertusa* is the dominating reef forming species. Its complex framework offers a multitude of different habitats [1], [2], [3] used by a great variety of species [4], [5], [6]. Among 1300 documented species [7] various symbiotic relations between scleractinian cold-water corals and associated invertebrates have been reported [8]. So far, most of these relationships are not clearly defined and their role in the functioning of the ecosystem is poorly understood [8], [9].

One ubiquitous species that is abundantly (12–17 ind. m^−2^, based on our data and the following references [10], [11]) observed in close contact with the cold-water coral *L. pertusa* is the polychaete *Eunice norvegica* [12], [13], [14]. It forms parchment-like tubes within living coral branches which later are calcified by its coral host [15]. Roberts [16] suggested that *E. norvegica* strengthens the reef framework by thickening and connecting coral branches. Moreover, by aggregating coral fragments the polychaete might enhance the development of large reef structures [16]. However, the relationship might come at a metabolic cost for the coral due to enhanced precipitation of CaCO_3._ So far however, no data is available to quantify this aspect of the relationship between coral and polychaete.

Additional to the indirect metabolic effect via calcification the polychaete also might have a more direct effect on coral metabolism: Aquaria observations by Mortensen [15] have shown that *E. norvegica* occasionally steals food from its host coral while at the same time it cleans the living coral framework from detritus and protects it from potential predators through aggressive territorial behavior. Again, the net outcome of these different processes on the metabolism of the coral and the polychaete has never been quantified.

Symbiosis are long term interactions between different biological species [17], [18] which can involve positive (mutualism (++), commensalism (+0)) and negative feedbacks (competition (--), parasitism (−+)) between the species [19], [20]. Based on qualitative observations the relations between *L. pertusa* and *E. norvegica* range from parasitic (food stealing) to mutualistic (cleaning and protection of coral branches) [15], [16]. However to better estimate the net outcome of the interplay of the different process involved quantitative data, especially with respect to species metabolism are necessary [21]. These data will further help to assess the significance of this interaction for the structure and functioning of cold-water coral reefs, which can be crucially influenced by species interactions [22].

In this study we directly quantify the interactions between *L. pertusa* and *E. norvegica* with respect to food assimilation, calcification and respiration, key processes in species metabolism and highly involved in the interaction between *L. pertusa* and *E. norvegica.* To trace and quantify these processes with respect to the interaction between both species we used two ^13^C and ^15^N labeled food sources that are considered important for cold-water coral reef communities, i.e. algae and zooplankton [23], [24], [25]. The use of two food sources also allowed us to investigate food competition and niche segregations between *L. pertusa* and *E. norvegica*. During the experiment corals and polychaetes were either kept separate or in association with each other to allow singling out the net outcome of the association on different metabolic processes. By using isotopically enriched food sources we were able to directly trace not only C and N tissue assimilation by *L. pertusa* and *E. norvegica,* but also calcification based on metabolically derived C-deposition in coral skeleton. Metabolically derived C, i.e. inorganic C originating from respired food, is one of the two sources that sustain the demand for inorganic C by calcification [26]. Since ^13^C-labeled food was used in our experimental design, the subsequent deposition of respired ^13^C into the coral skeleton was used as proxy for calcification as discussed below.

## Materials and Methods

### Sampling Location and Maintenance

All coral pieces and polychaetes used in the experiments were obtained from the Norwegian Tisler Reef, with all necessary permits obtained from the Directorate of Fisheries, Norway to conduct the described study. The Reef is situated at 70 to 155 m depth in the NE Skagerrak, close to the border between Norway and Sweden. Throughout the year, the current velocity over the reef normally varies from 0 to 50 cm s^−1^, with peaks in excess of 70 cm s^−1^, while the flow direction fluctuates irregularly between NW and SE [27], [28]. Temperature at the reef site typically varies between 6 to 9°C throughout the year [27], [28]. The amount and quality of particulate organic carbon (POC) reaching the reef depends on the location within the reef, so that POC concentrations can range from 43.5 to 106.3 µg C L^−1^ [28].

Specimens were collected from a depth of around 110 m (N58°59,800' E10°58,045') with the remotely operated vehicle (ROV) Sperre Subfighter 7500 DC. Within a few hours they arrived in the laboratory at the Sven Lovén Centre in Tjärnö (Sweden) in cooling boxes filled with Koster-fjord seawater (7°C, salinity 31). Before used in the experiment organisms were kept 1–8 days for acclimation in a dark temperature-controlled laboratory at 7°C with sand-filtered running seawater from 45 m depth out of the adjacent Koster-fjord (sand particle size 1–2 mm, water exchange rate of ca 1 L min^−1^). No additional feeding was offered during the maintenance period since the sand-filtered Koster-fjord water still contained a lot of particles <1–2 mm (pers. observation) which could be used as food source [24]. During the acclimation phase corals were kept without polychaetes, while polychaetes were kept in aquaria with coral rubble including also living colonies in response to their need of shelter. One day before used in the experiment larger coral colonies were clipped to approximately the same size, determined by dimension and buoyant weight, to allow using comparable coral samples per chamber. Polychaetes were selected solely on the base of their dimension. After the experiment all samples were also measured for dry weight (DW) to allow standardization among treatments.

### Preparation of Food Substrates

Cold-water corals are exposed to various food particles and they are considered to feed on a mixed diet including phyto- and zooplankton [23], [25], [29], [30]. The diatom *Thalassiosira pseudonana* (5 µm) was chosen to represent small phytoplankton-derived particulate organic matter (POM) substrates reaching cold-water coral reefs [25]. They were cultured axenically in f/2 medium adapted after Guillard [31] on the base of artificial seawater in which 90% of either the unlabeled NaHCO_3_ or NaNO_3_ were exchanged with the isotopically enriched equivalent (Cambridge Isotopes, 99% ^13^C, 99% ^15^N). After 3 weeks of culturing cell densities were around 3–4*10^6^ cells ml^−1^ and the algal suspension was concentrated by centrifugation at 450 g. The concentrates were rinsed three times with 0.2 µm filtered seawater to remove residual label. The isotopic enrichment of the algal concentrates were 59% ^13^C (δ^13^C 125908 ‰) and 64% ^15^N (δ^15^N 472590 ‰), respectively. They were stored frozen until use in the experiment (for details on isotope analysis, see below).

*Artemia spp.* nauplii (∼300 µm) were chosen to represent large zooplankton-derived POM substrates reaching cold-water coral reefs [23]. They resemble natural zooplankton and have been successfully used in food studies on *Lophelia pertusa* before [32], [33], [34]. ^13^C and ^15^N enriched nauplii were cultured by hatching 0.6 g *Artemia* cysts (Sera) in 10 L incubation chambers filled with 0.2 µm filtered seawater under natural light conditions and mild aeration. After the larvae had developed to a state at which they take up particulate food (1 to 2 days after eclosion of larvae), larvae were fed every second day with a suspension containing ^13^C or ^15^N enriched pre-cultured algae (at around 1.5 mg C L^−1^ and 0.15 mg N L^−1^ respectively). The uptake of algae was confirmed visually under the microscope as green food particles in the animal guts. After seven days of feeding, larvae were concentrated by filtration on a 200 µm filter, rinsed off the filter with filtered seawater, counted under the binocular and stored frozen. Within the *Artemia* concentrate different early larval stages could be detected. The final isotopic enrichment of the larvae was 4% (δ^13^C 2909 ‰) for ^13^C and 7% (δ^15^N 21800 ‰) for ^15^N respectively.

To standardize the amount of C added to the incubations, substrates were analyzed for C and N content (see below for methodological description). The 1∶1 mixture of ^13^C Artemia : ^15^N algae and ^13^C algae : ^15^N *Artemia*, both at total concentration of 800 µg C L^−1^, represented the two food treatments. Together with the treatments “*Lophelia* separate”, “*Eunice* separate” and “*Lophelia* - *Eunice* together” gives a total of 6 treatments, each of them performed in triplicate.

### Experimental Set up and Procedure

**Figure 1:**
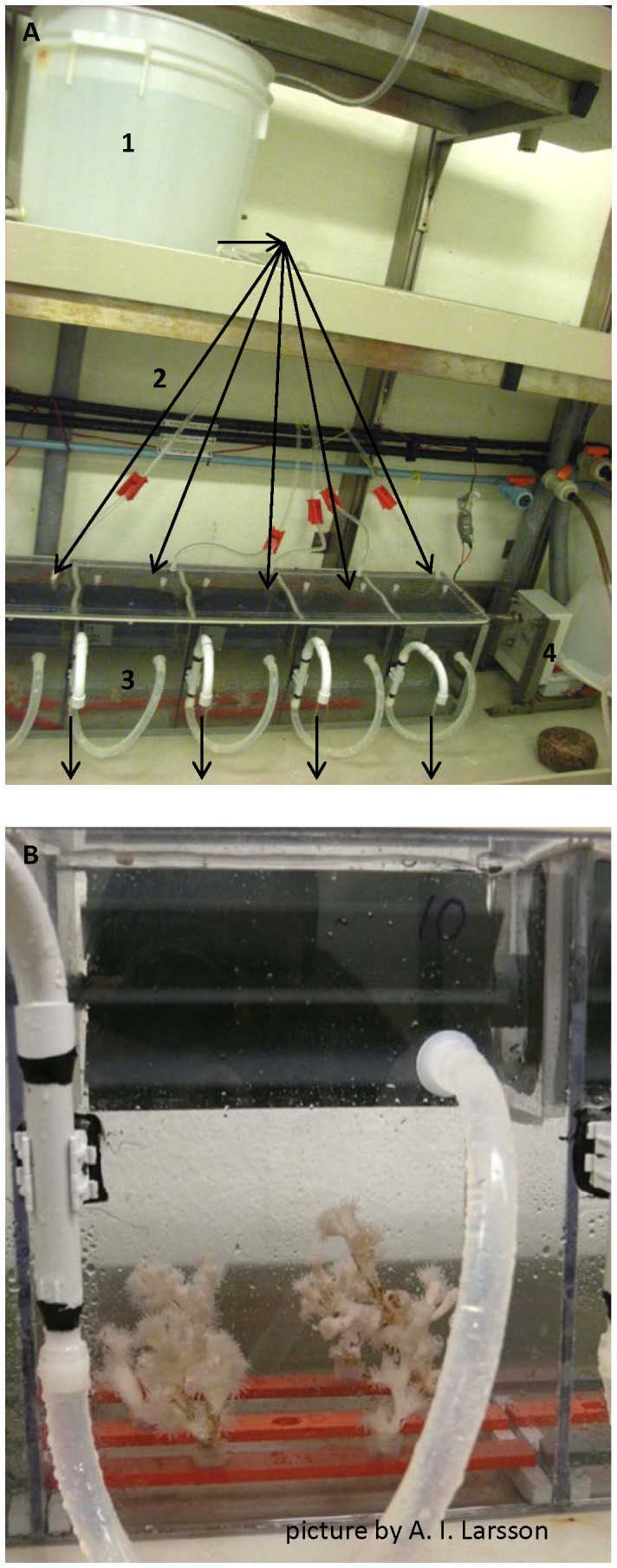
Experimental set up (Mississippi chambers). a) Experimental set up including the water reservoir for filtered sea water (1), the direction of the water flow indicated with arrows (2), 5 incubation chambers (3) and the motor driving the chamber paddles (4). b) Close-up of one incubation chamber (10 L) provided by A. I. Larsson.

Incubation chambers (Fig. 1) were placed in a thermo-controlled room at 7°C and filled with 5 µm filtered Koster-fjord bottom water prior to the start of the experiment. A total of 29 coral fragments (2 to 3 coral pieces chamber^−1^, 3.2±1.33 g DW piece^−1^; 9.78±1.16 g DW chamber^−1^; 16.55±5.43 polyps chamber^−1^, with no significant difference of coral weight between treatments, p>0.05) and 12 polychaete specimens (1 polychaete chamber^−1^, 0.48±0.12 g DW polychaete^−1^, with no significant difference of polychaete weight between treatments, p>0.05) were selected and placed separately or together in the middle of the incubation chambers. To stabilize the corals fragments in an upright position, they were gently inserted into a 1 cm elastic silicone tube on an acrylic plate that was attached on the chamber base. To provide refuge space in treatments with only polychaetes present bleached coral skeleton and plastic tubes were placed in the chambers. Both substitutes were indeed used as refuge by the polychaetes during the experiment, although the tubes were not present during the acclimation phase. Chambers with corals and polychaetes contained only living coral specimens, used as shelter by the polychaete during the incubation. Water circulation was maintained during the experimental period by a motor-driven paddle in the upper part of the incubation chamber (Fig. 1, rotor speed 30 rpm). The motor section was not directly attached to the incubation chamber and effectively avoided heating of the chambers during the incubations. Corals and polychaetes were left in the chamber for 12 h for acclimatization prior to feeding.

At the start of the experiment, 400 µg C L^−1^ of each food source was gently pipetted into the water column of each chamber. The chambers were closed from flow through for 2.5 days to allow feeding (feeding period). Visual observation confirmed that the circulating water kept the food particles in suspension. After the feeding period, the chambers were flushed to remove remaining food particles and waste products by pumping 5 µm filtered Koster-fjord bottom water through the chambers at a flow speed of 140 ml min^−1^ for 12 h. This pattern was conducted twice. After the last flushing period (140 ml min^−1^, 5 µm filtered Koster-fjord water, lasting 12 h), incubation chambers were closed without food addition for another 24 h. During this period the respiration of the added ^13^C enriched food substrates was quantified by measuring the production of dissolved inorganic ^13^C in the water (^13^C-DIC) [35], [36], [37]. Water samples were taken before (control) and after the respiration incubation, and filtered (GF/F) in a 20 ml headspace vial. Each sample was poisoned with 10 µl HgCl_2_, closed with an aluminum cap fitted with a rubber septum and stored upside down.

In parallel to the main experiment, 3 control corals and 3 control polychaetes were incubated without food for stable isotope ^13^C and ^15^N background measurements. After a total experimental time of 7.5 days, coral and polychaete samples were frozen at −20°C and transported to the Netherlands Institute for Sea Research-Yerseke, where they were freeze-dried and stored frozen for further analysis.

### Sample Treatment and Analysis

#### Tissue assimilation

Prior to isotopic analysis frozen coral and polychaete samples were freeze-dried, weighed and homogenized by grinding with a ball Mill for 20 s (MM 2000, Retsch, Haan, Germany). A subsample of around 30 mg of grinded coral material and 2–3 mg grinded polychaete material was transferred to pre-combusted silver boats and decalcified by acidification. While polychaete samples were directly acidified with concentrated HCl (12 mol L^−1^), coral samples were first placed in an acidic fume for 3 to 4 days to remove most of the inorganic C. Coral samples were then further acidified by stepwise addition of HCl with increasing concentration (maximum concentration 12 mol L^−1^) until the inorganic C fraction (skeleton) was fully removed (as evidenced by the absence of bubbling after further acid addition). The remaining fraction after acidification resembled the organic fraction of each samples, which in case of the coral samples includes the coral tissue and the organic matrix in the skeleton that represents only a very small organic fraction [38]. After complete decalcification each sample was measured for ^13^C and ^15^N using a thermo Electron Flash EA 1112 analyzer (EA) coupled to a Delta V isotope ratio mass spectrometer (IRMS).

All obtained stable isotope data were expressed in µg C g^−1^ C biomass and µg N g^−1^ N biomass. They were calculated as following on the base of the delta notations obtained from the IRMS: δX (‰) = (R_sample_/R_ref_ −1)*1000, where X is the element, R_sample_ is the heavy : light isotope ratio in the sample and R_ref_ is the heavy : light isotope ratio in the reference material (Vienna Pee Dee Belemnite standard for C and atmospheric nitrogen for N). When used for C the R_ref_  = 0.0111797 and when used for N then R_ref_  = 0.0036765. The atomic % of heavy isotope in a sample is calculated as F = R_sample_/(R_sample_+1). The excess (above background) atm % is the difference between the F in an experimental sample and the atm % in a control sample: E = F_sample_ – F_control_. To arrive to total tracer C and tracer N uptake of labeled food substrates, the excess incorporation was divided by the atm % of each specific food source.

#### Calcification (tracer C incorporation in coral skeleton)

To measure the incorporation of tracer C in coral skeleton 30 mg of each coral sample (including tissue, organic matrix and skeleton) was directly transferred to a silver boat and measured on the EA-IRMS for total ^13^C content. The same calculations used to calculate tracer C tissue assimilation were then used to calculate tracer C incorporation in the total C pool of the coral sample. Incorporation of tracer C in the inorganic skeleton was finally determined by subtracting tracer C assimilation in the organic C fraction (tissue and organic matrix) from the tracer C incorporation in the total C pool (tissue, organic matrix and skeleton). This allowed us to trace the metabolically derived tracer C, i.e. from food respiration, into the coral skeleton and to quantify calcification rates based on this C source. Although this calcification process may be of limited importance to total calcification by cold water corals [39], a comparison can reveal calcification differences between treatments. Another advantage is that small changes within a small time period can be detected, similar to the ^45^Ca labeling method [40], [41], but without the necessity of radioactive isotopes.

#### Respiration

Respiration of labeled food substrates was measured by analyzing the concentration and isotopic ratio of the CO_2_ in the water samples taken at the beginning and the end of the 24 h incubation at the end of the experiment. After creating a headspace of 3 ml in each sample vial by injecting N_2_ gas through the vial septum [36], [42], samples were acidified with 20 µl of concentrated H_3_PO_4_ to transform DIC into CO_2_. After CO_2_ had exchanged with the vial headspace 10 µl sample of the headspace gas was injected into an elemental analyzer isotope-ratio mass spectrometer (EA-IRMS). The final calculations for tracer C respiration followed the description for tracer C tissue assimilation.

### Statistics

The program PERMANOVA [43] was used to investigate interactions between the different factors (food and treatment) by permutational multivariate analysis of variance (PERMANOVA). The outcome of each PERMANOVA test was expressed in Monte Carlo P-values, which are more robust in case of smaller numbers of replicates. If the variance between data was not homogeneous (tested using Fligner-Killeen test) a Kruskal-Wallis test was used as true non-parametric approach.

## Results

### Tissue Assimilation and Respiration by Eunice Norvegica

**Figure 2:**
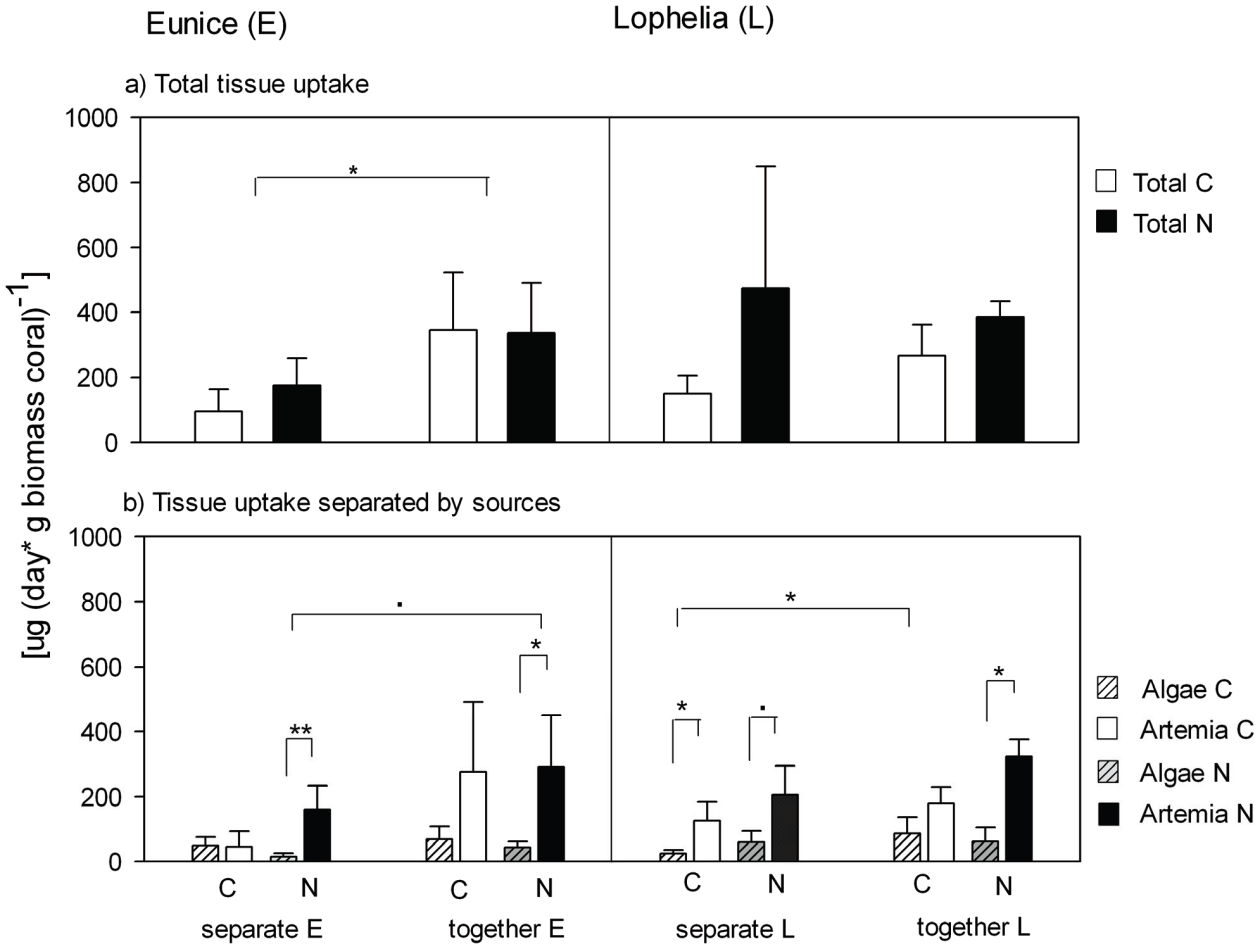
Tissue uptake by *E. norvegica* (E) and *L. pertusa* (L.). a) Total tracer C and tracer N tissue uptake, b) Tracer C and tracer N tissue uptake separated by food substrate (algae, *Artemia*). Animals were incubated separate or together in one chamber; statistical significance between treatments is indicated as followed: **p<0.009, *p<0.05, · 0.05<p<0.06. The bars in each figure represent average ± SD.

*E. norvegica* assimilated in total 95±67 µg C (day * g C biomass polychaete)^−1^ and 175.51±83.05 µg N (day * g N biomass polychaete)^−1^ in the absence of *L. pertusa* (Fig. 2a). However when *L. pertusa* was present, the assimilation of *E. norvegica* was significantly enhanced 4 times for C and 2 times for N (PERMANOVA p  = 0.03, Fig. 2a).

C partitioning between different food sources did not differ significantly among treatments (PERMANOVA p≥0.1), although a trend of higher *Artemia* uptake in the presence of the coral was recorded (Fig. 2b). However in both, absence and presence of corals, *Artemia* was the dominant N-source for *E. norvegica*, accounting for 87% of total assimilated N in polychaete tissue when corals were present to 91% when corals were absent (PERMANOVA p≤0.02, Fig. 2b).

**Figure 3:**
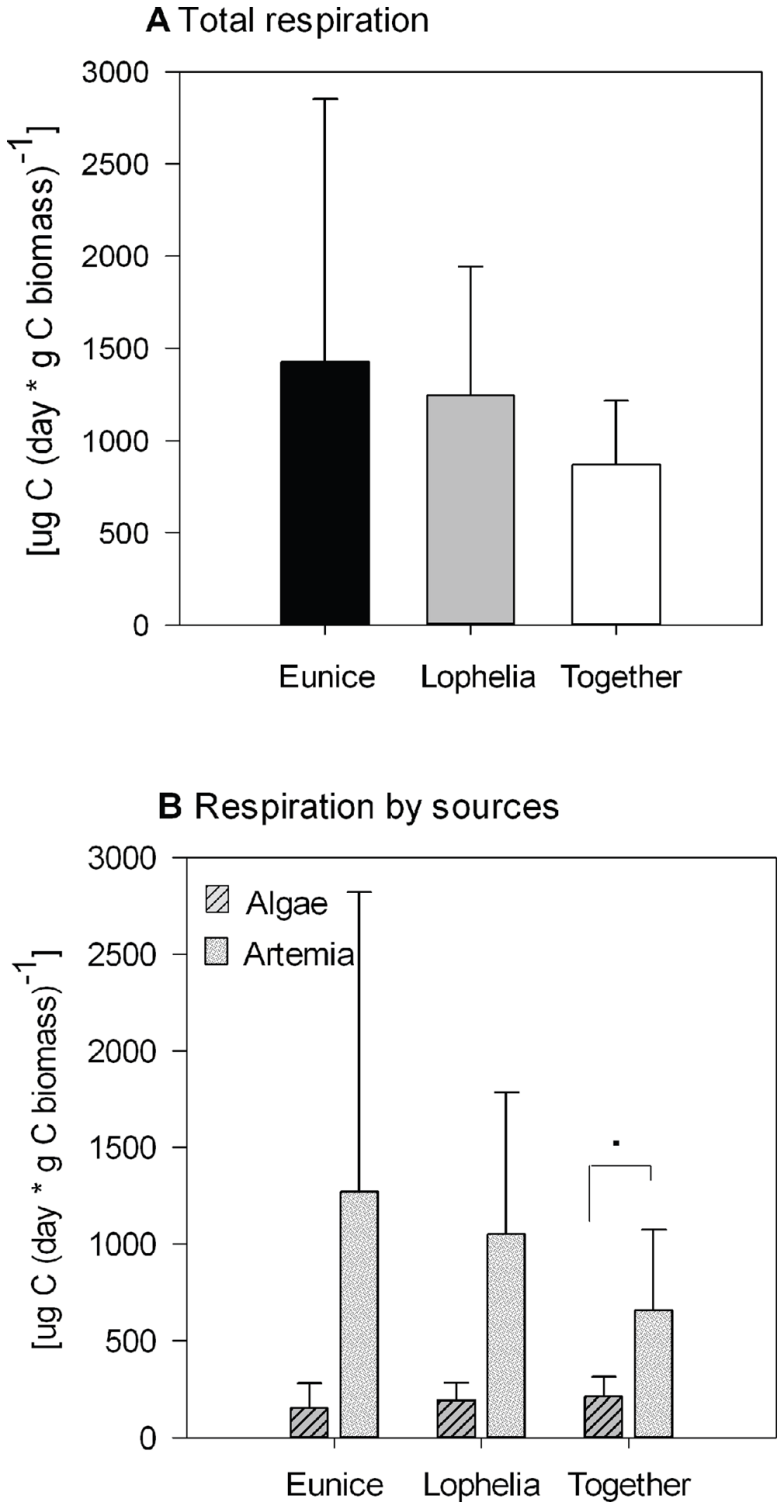
Respiration by *E. norvegica* (E) and *L. pertusa* (L.). a) Total tracer C respiration, b) tracer C respiration separated by food substrate (algae, *Artemia*). Animals were incubated separate or together in one chamber; statistical significance between treatments is indicated as followed: **p<0.009, *p<0.05, · 0.05<p<0.06. The bars in each figure represent average ± SD.

During incubations without corals, *E. norvegica* respired 1423±1431 µg C (day * g C biomass polychaete)^−1^. Most of the C respired by the polychaete in these incubations was derived from its feeding on *Artemia* (Fig. 3a). Total respiration in incubations with only the polychaete present did not differ from incubations with *E. norvegica* and *L. pertusa* present (PERMANOVA p  = 0.7, Fig. 3a).

### Tissue Assimilation, Calcification and Respiration by Lophelia Pertusa

In total *L. pertusa* assimilated 149±56 µg C (day * g C biomass coral)^−1^ and 473±375 µg N (day * g N biomass coral)^−1^ when *E. norvegica* was absent (Fig. 2a). Neither the uptake of C nor the uptake of N was significantly affected by the presence of the polychaete (PERMANOVA p  = 0.3, Fig. 2a). Even though the presence of *E. norvegica* did not change total C and N tissue assimilation of *L. pertusa,* it did change the contribution of food sources (Fig. 2a, b). In the absence of the polychaete *L. pertusa* significantly assimilated more *Artemia* then algae (PERMANOVA p  = 0.02, Fig. 2b). In the presence of *E. norvegica* however *L. pertusa* increased the assimilation of algal-derived C from 16% to 33% (PERMANOVA p  = 0.07, Fig. 2b), which resulted in equal assimilation rates of both food sources (PERMANOVA p  = 0.1, Fig. 2b). This trend was not visible for N assimilation, where no significant influence by *E. norvegica* on food utilization by *L. pertusa* could be observed (PERMANOVA p  = 1) and *Artemia* remained the dominant source of N for the coral independently of *E. norvegica* presence or absence (PERMANOVA p≤0.5, Fig. 2b).

**Figure 4:**
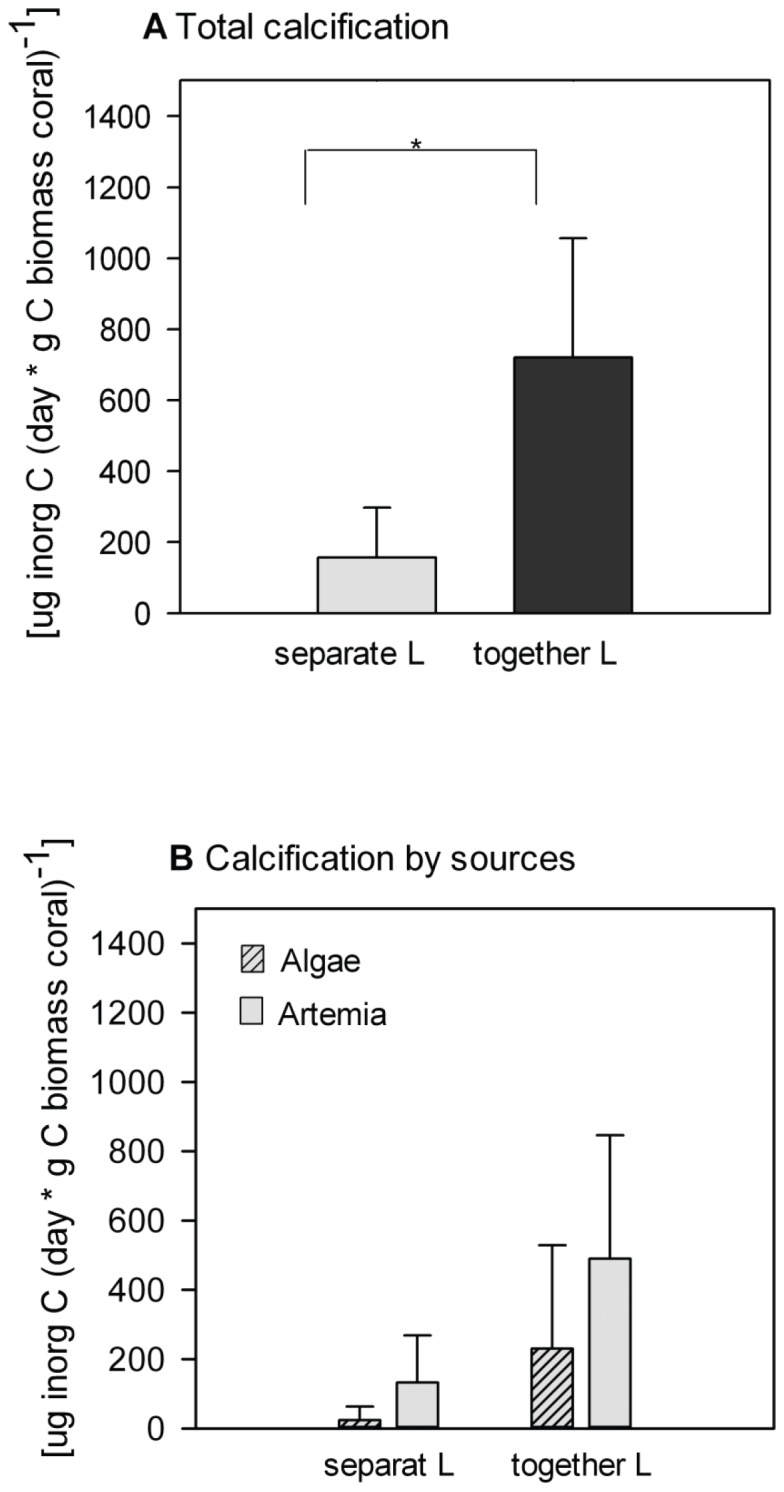
Calcification by *L. pertusa.* a) Total calcification, b) calcification separated by food substrate (algae, *Artemia*). *L. pertusa* was kept with and without *E. norvegica*; statistical significance between treatments is indicated as followed: ** p<0.009, * p<0.05, · 0.05<p<0.06. The bars in each figure represent average ± SD.

Metabolic derived coral calcification was significantly enhanced up to 4 times by the presence of the polychaete (Kruskal-Wallis p  = 0.05, Fig. 4a). On average, *Artemia* contributed 68% and 85% to total inorganic C formation, whereas algae accounted for 32% and 15% in absence and presence of the polychaete respectively (Fig. 4b). These contributions were however not significantly different (Kruskal-Wallis p = 0.2, Fig. 4b).

Coral respiration in the absence of *E. norvegica* accounted for 1242±699 µg C (day * g C biomass coral)^−1^. *Artemia* was the primary C source and supplied 85% of the respired C, in contrast to algae, which contributed only 15% (Fig. 3a, 3b). Respiration in the absence of the polychaete was not significantly different from incubations where *E. norvegica* was present (PERMANOVA p  = 0.5, Fig. 3a).

### C budget of Lophelia Pertusa and Eunice Norvegica Separately and Together

**Figure 5:**
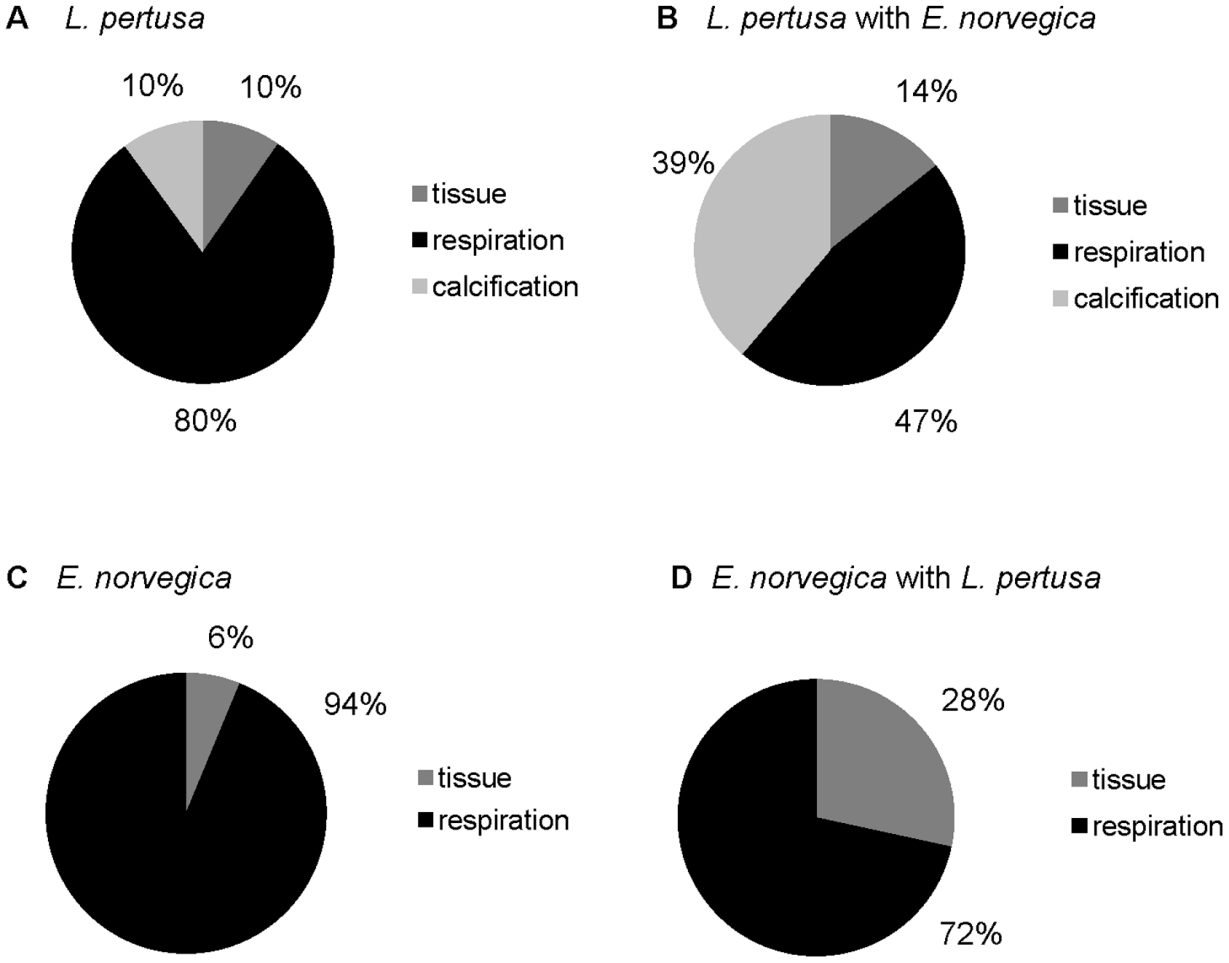
Carbon budget of *L. pertusa* and *E. norvegica*. (a) C-budget for *L. pertusa* with and without *E. norvegica*, b) C-budget for *E. norvegica* with and without *L. pertusa*. Each budget is based on total tracer recovery. The partitioning between tissue assimilation, respiration and calcification are expressed relatively to total tracer C uptake (sum of assimilation, respiration and calcification).

Partitioning of tracer C between tissue assimilation, calcification and respiration was merged into a C budget for each treatment based on total tracer C tracer uptake by *L. pertusa* and *E. norvegica*. This revealed that in the absence of *E. norvegica, L. pertusa* invests 10% of total acquired C in tissue, 10% in calcification and 80% in respiration (Fig. 5a, Table 1). With *E. norvegica* present, however, this picture changed, mainly due to higher calcification rates stimulated by the presence of the polychaete. On average 14% of total acquired C was transferred into tissue, 39% was recovered in carbonate and 47% lost by respiration (Fig. 5b, Table 1).

**Table 1:** Tracer C-partitioning between metabolic components of *L. pertusa* and *E. norvegica* separate and together. (n = 3).

Component	*L. pertusa* *	*L. pertusa+E. norvegica* *	*E. norvegica* *	*E. norvegica+L. pertusa* *
Tissue	150±56	266±95	95±68	345±177
Respiration	1243±700	869±345	1423±1431	869±45
Calcification	156±141	720±336		

* [µg C (day*g C biomass)^−1.^

For *E. norvegica,* the main change in the C budget was higher food assimilation in the presence of the coral. When *L. pertusa* was absent *E. norvegica* assimilated 6 of total acquired C in the tissue while 94% of total acquired C was lost by respiration (Fig. 5c, Table 1). With *L. pertusa* present *E. norvegica* increased its tissue C-uptake up to 28% while only 72% of acquired C was used for respiration (Fig. 5d, Table 1).

## Discussion

### Assimilation and Calcification in the Symbiotic Coral-polychaete Relation

#### Eunice norvegica

In this study we quantified the qualitative observations of the interaction between *E. norvegica* and *L. pertusa* to infer the importance of this interaction for cold-water coral ecosystems. Results revealed that *Eunice* assimilated 4 times more C and 2 times more N in the presence of the coral. Respiration however was independent of coral presence but well within the range of former observations [10]. The polychaete further tended to switch to a more selective food uptake, preferentially taking up bigger particles when *L. pertusa* was present (Fig. 2b). These results are in agreement with previous behavioral observations, where *E. norvegica* has been reported to steal mainly bigger food items from its coral host [15]. We hypothesize that larger particles cause longer handling times by the coral during the process of feeding [44], [45], which gives the polychaete more time to remove these particles from the polyp surface. Small particles might be consumed faster by the coral host and also might be more effectively anchored within the mucus layer of the coral [45], [46], [47]. Since mucus is used by the coral not only to trap, but also to transport particles to its mouth, [48], [49], a weaker binding of larger particles within the coral mucus layer would make it easier for the polychaete to access and remove those from the coral surface.

The observed high influence of coral presence on polychaete nutrition evidences that the interaction not only provides settlement and shelter but also increases the fitness of the polychaete. Coral rubble and dead framework can also provide shelter, but they are neither able to help in tube strengthening by calcification nor in food supply, since the coral is dead. The increased food input by proximity to living corals might explain the common occurrence of *E. norvegica* within living coral branches as one of two species so far documented living in direct contact to coral tissue [11]. The advantage of living within the live coral becomes even clearer with regard to the location of the tube selected by the polychaete and its reef aggregating behavior described by Roberts [16]. To ensure its benefits, the polychaete places its tube openings close to big coral polyps [2], [15] and moves small broken coral branches within reach of its tube [16].

#### Lophelia pertusa

In contrast to *E. norvegica* total C or N uptake by *L. pertusa* was not influenced by the presence of the polychaete. Instead, *L. pertusa* switched from preferential feeding on *Artemia* to more opportunistic feeding by enhancing the uptake of smaller particles in the presence of the polychaete. The higher contribution of smaller particles in the presence of the polychaete is most likely caused by the preferential stealing of bigger particles by the polychaete (see above), leaving the coral to feed on what is left over. This implies that the success of *L. pertusa* to exploit a certain food source depends not only on the availability of the source but also on the interference with other species living in close association with the coral.

While experiments conducted on *L. pertusa* in isolation will help to understand its capabilities and potential, interactions between species have to be elucidated to advance our understanding of the species in the context of its natural environment. The interaction with *E. norvegica* here implies that laboratory studies done only with corals and large food particles, might overestimate the importance of these sources for the coral in its natural environment. However, the ability to utilize a broad range of food sources probably ensures that *L. pertusa* does not suffer from stealing by *E. norvegica*, as indicated by the observation that total assimilation and respiration were not affected by the polychaetes’ presence. We further show that calcification by corals increased in the presence of the polychaete up to 4 times compared to treatments with only corals present, confirming the assumption that the coral enhanced calcification while interacting with *E. norvegica* [16]. Hence, this interaction may influence total calcification in a reef and therefore reef development. The coral pieces in our study showed no previous impact of polychaete presence like old tube remains, polyp-malformation or thickening. This indicates that the observed higher calcification rate is related to the initial phase of the polychaete-coral relationship during the polychaetes’ tube formation. It is however likely that the positive feedback on calcification continues during the entire coral-polychaete relationship, since the polychaete keeps on elongating and rearranging its tube around coral branches with time [16].

Surprisingly, however, this enhanced calcification was not accompanied by higher metabolic activity represented by total respiration. Naumann et al. [32] found that changes in calcification in the cold-water coral *Desmophyllum dianthus* are correlated to changes in respiration.

In contrast to that however and in agreement with our observations Form & Riebesell [50] found that enhanced calcification by *L. pertusa* under high CO_2_ exposure did not entail enhanced respiration. This implies species-specific differences in calcification as suggested by Adkins et al. [39] resulting in a more conservative calcification by *L. pertusa* than by *D. dianthus*.

### Implications for Ecosystem Functioning

Cold-water coral reefs have been described as hotspots of C cycling [10] and biodiversity along continental margins [5], [6], [9]. So far, most aquaria studies focused on individual key species within the system, in particular the cold-water coral *Lophelia pertusa* [33], [51], [52]. Here we evidently show that interactions between species may substantially contribute to the development and functioning of a reef. Apparently, not only competition between species, but also facilitation can shape ecosystems by cascading throughout the community and so affect ecosystem functioning [22], [53], [54] and persistence, especially under changing environmental conditions [55].

In this study we showed that *Eunice norvegica* positively influences coral calcification and changes food partitioning, however without impacting total energy uptake by its coral host. So far, calcification of cold-water corals has been studied in an isolated single-species setting and quantified in the context of environmental changes ([56] and references therein), but knowledge on the influence of biological interactions is limited and qualitative [57], [58]. Our results suggest, however, that the importance of biological interactions for the process of calcification in a reef environment might have been underestimated, since we measured a 4 times increase of calcification when interaction between *L. pertusa* and *E. norvegica* could take place. Enhanced calcification results in branch thickening and anastomosis, which facilitates reef growth and framework strength and thus can enhance ecosystem development and persistence, since the development of coral skeleton is essential for this ecosystem [16].

It is however yet unclear how this interaction is affected by changing environmental conditions, such as ocean acidification and warming, or how this interaction reflects upon the impact of such changes on reef development. To improve our prediction of the future of cold-water coral reefs it is not only necessary to study the coral itself under various conditions but also to account for the many organisms living in association with the coral and contributing to the formation of this unique ecosystem.
